# MicroRNAs at the crossroad of cancer therapeutics: insights from WNT signaling & flavonoids

**DOI:** 10.3389/fmolb.2025.1616221

**Published:** 2025-08-12

**Authors:** Akanksha Gupta, Arpit Mehrotra, Abhilasha Sood, Bunty Sharma, Vikas Yadav, Ginpreet Kaur, Katrin Sak, Shakti Ranjan Satapathy, Hardeep Singh Tuli

**Affiliations:** ^1^ Department of Pharmacology, Shobha Ben Pratap Bhai Patel School of Pharmacy and Technology Management, SVKM’S NMIMS, Mumbai, India; ^2^ Department of Allied Health Sciences, Chitkara School of Health Sciences, Chitkara University, Rajpura, Punjab, India; ^3^ Department of Biotechnology, Graphic Era (Deemed to be University), Dehradun, Uttarakhand, India; ^4^ Cell and Experimental Pathology, Department of Translational Medicine, Skåne University Hospital, Lund University, Malmö, Sweden; ^5^ NGO Praeventio, Tartu, Estonia; ^6^ Centre of Excellence in Computational Research and Drug Discovery, Department of Bio-Sciences and Technology, Maharishi Markandeshwar Engineering College, Maharishi Markandeshwar (Deemed to be University), Mullana, India

**Keywords:** wnt signaling, microRNA, cancer, flavonoids, apoptosis, metastasis

## Abstract

MicroRNAs (miRNAs) are pivotal post-transcriptional regulators that orchestrate gene expression programs governing cancer initiation, progression, metastasis, and therapeutic resistance. Among their many targets, the WNT signaling pathway, a key driver of malignancy, is tightly controlled by miRNAs, forming intricate feedback loops that shape tumor behavior. Concurrently, flavonoids, naturally occurring plant-derived polyphenols, are emerging as promising anticancer agents that can modulate both WNT signaling and miRNA expression. This review highlights miRNAs as the central regulators of oncogenic signaling, focusing on their dualistic role in cancer biology and their modulation by flavonoids. We explore the mechanistic frameworks underpinning miRNA-WNT interactions and the therapeutic potential of flavonoid-mediated miRNA reprogramming for precision miRNA targeting. Unraveling this regulatory axis offers a promising avenue for developing multi-targeted therapies and personalized cancer treatment strategies.

## 1 Introduction

Despite decades of research, cancer continues to be one of the leading causes of death globally, claiming nearly ten million lives each year (Bray et al., 2024). The transformation from a single mutated cell into an invasive malignant tumor typically spans 10–20 years, eventually progressing into a systemic disease that affects the entire body (Loeb and Harris, 2008). Metastasis and resistance to therapy are the primary drivers of cancer-related mortality, posing a major clinical and socioeconomic challenge (Kilmister et al., 2022; Emran et al., 2022). Consequently, there is an urgent need for innovative treatment strategies that can curb tumor growth, prevent its spread, and efficiently target rapidly dividing cancer cells.

MiRNAs (microRNAs) have evolved into vital post-transcriptional gene expression regulators and modulators of cell function in response to signaling molecules over the past few years. MiRNAs are evolutionarily conserved, small non-coding RNAs (∼22 nucleotides) that fine-tune gene expression at the post-transcriptional level. Their dysregulation plays a fundamental role in the pathogenesis of various cancers, including colorectal, breast, and hepatocellular carcinomas, by affecting critical processes such as cell proliferation, epithelial-mesenchymal transition (EMT), angiogenesis, metastasis, and therapy resistance. MiRNAs interact with essential cellular signaling pathways, including the WNT, TGF-β, and Notch pathways. They regulate stem cell activity to maintain tissue homeostasis; however, when they become dysregulated, they can contribute to the onset and progression of cancer (Li H. et al., 2024; Talebi et al., 2023; Onyido et al., 2016). Among the diverse signaling cascades influenced by miRNAs, the WNT signaling pathway emerges as one of the most frequently targeted and reciprocally modulated signaling pathways in cancer biology.

WNT signaling orchestrates key developmental and homeostatic events; however, its aberrant activation, often driven by mutations in *APC*, *CTNNB1*, or *AXIN*, significantly contributes to the development of cancer. It regulates malignant growth and influences drug resistance, making it an attractive target for novel cancer therapies (Hushmandi et al., 2025; Groenewald et al., 2023; Yadav et al., 2021). The activation of WNT signaling can lead to the loss of normal cell polarity and adhesion, thereby facilitating tumor progression, invasion, and metastasis in various human cancer malignancies (Hushmandi et al., 2025; Groenewald et al., 2023; Yadav et al., 2021). This signaling pathway is well known to be post-transcriptionally regulated by non-coding RNAs, including miRNAs, which can suppress or enhance the expression of WNT cascade-related genes in different cancer types (Onyido et al., 2016; Lei et al., 2020). An increasing body of evidence highlights miRNAs as crucial modulators of this pathway, either by directly targeting WNT components or being transcriptionally regulated by WNT-driven transcription factors like TCF/LEF. This bidirectional regulatory loop has profound implications for tumor behavior and therapeutic response. Moreover, high-throughput sequencing and omics-based technologies have significantly enhanced the fundamental comprehension of miRNA-mediated regulation of the WNT-signaling pathway and vice versa. Therefore, evidence suggests that molecules targeting this WNT-signaling/miRNAs complex are a better therapeutic possibility for restricting cancerous growth.

In parallel, dietary polyphenols, especially flavonoids such as quercetin, apigenin, and baicalein, have garnered attention for their epigenetic and post-transcriptional regulatory properties (Sak, 2014; Pyo et al., 2024; Tuli et al., 2023). These naturally occurring compounds modulate miRNA expression profiles, thereby indirectly influencing oncogenic pathways, such as the WNT pathway, and exhibit potent anti-proliferative, pro-apoptotic, and anti-metastatic activities. Their dual capacity to influence both miRNAs and downstream signaling pathways positions flavonoids as promising agents in integrative cancer therapy.

This review comprehensively synthesizes recent findings on the miRNA-WNT-flavonoid triad in cancer. Unlike previous reviews, which often focus separately on either WNT signaling, miRNA regulation, or flavonoid effects, this review uniquely integrates these components into a cohesive therapeutic triad. We specifically address how flavonoids modulate the complex interplay between miRNAs and WNT signaling, highlighting novel therapeutic opportunities. We first explore how specific miRNAs regulate WNT signaling and contribute to cancer progression. We then examine how flavonoids modulate these miRNAs and discuss their therapeutic implications. Finally, we highlight emerging strategies, including nano-delivery systems and omics-based miRNA profiling, to translate these insights into precision oncology frameworks.

## 2 WNT signaling and its intricate relationship with miRNAs in cancer

To understand the role of flavonoids as modulators, it is first necessary to appreciate the complexity of the WNT signaling pathways and how miRNAs act as key regulators within this system. This section establishes the molecular foundation of how WNT signaling is regulated in both canonical and non-canonical forms, highlighting the bidirectional crosstalk between miRNAs and WNT components that contributes to cancer progression.

### 2.1 WNT signaling pathway and its role in cancer

WNT signaling represents a highly specialized biochemical cascade and a complex communication network regulating cell fate decision-making, tissue homeostasis, and carcinogenesis (Hushmandi et al., 2025; Groenewald et al., 2023; Yadav et al., 2021). Its ubiquitous role in development makes it foundational in all cell types, but its dysregulation leading to cancer makes it paradoxically a double-edged sword in the cellular biology field. Initially well-defined, its numerous complexities have since expanded, revealing non-canonical links to metabolic reprogramming, immune evasion, and cellular plasticity within the tumor microenvironment. The WNT pathway is divided into canonical (β-catenin-dependent) and non-canonical (β-catenin-independent) branches, dictating distinct oncogenic pathways (Yadav et al., 2021; Clevers and Nusse, 2012; Yadav et al., 2023).

#### 2.1.1 Canonical and non-canonical WNT signaling pathway

Under homeostatic conditions, β-catenin is stringently regulated via a destruction complex containing adenomatous polyposis coli (APC), AXIN, glycogen synthase kinase-3β (GSK-3β), and casein kinase 1 (CK1) (Zhan et al., 2017). In the presence of WNT ligands, β-catenin is phosphorylated and subsequently degraded through the proteasome, thereby preserving cellular homeostasis. In cancer, mutations in *APC* or *CTNNB1* (β-catenin) lead to permissive β-catenin machinery, subverting TCF/LEF transcription factors to unleash tumorigenic gene expression, including *MYC*, *CCND1*, and *AXIN2*, promoting unbridled proliferation, stemness, and therapeutic resistance. Colorectal cancer (CRC) is a prototypical example of canonical WNT hyperactivation, and mutations of the APC tumor suppressor are found in ∼approximately 80% of sporadic CRC cases. These mutations lead to the production of truncated forms of APC, which are unable to sequester β-catenin, thereby resulting in the constitutive activation of the WNT pathway. However, this oncogenic monopoly is not universal; *CTNNB1* mutations often replace *APC* loss in hepatocellular carcinoma (HCC) and medulloblastoma, highlighting the context-dependent nature of WNT perturbation (Zhao et al., 2022; Nie et al., 2020).

Non-canonical WNT pathways operate via β-catenin-independent means and can be broadly categorized into planar cell polarity (PCP) and WNT/Ca^2+^ pathways. These projections are critical in cytoskeletal reorganization, cell mobility, and microenvironment adaptation, which may promote metastasis. In the PCP Pathway, WNT ligands such as WNT5A and WNT11 bind to Frizzled (FZD) and receptor tyrosine kinase-like orphan receptors (ROR1/2), activating Rho GTPases (RhoA, Rac1, Cdc42) (Groenewald et al., 2023). This signaling cascade regulates actin polymerization, extracellular matrix remodeling, and directional migration. Several cancer types, including breast, prostate, and melanoma, exploit this mechanism through WNT5A-ROR2 signaling, which promotes invasive phenotypes and contributes to resistance against targeted therapies (O'Brien et al., 2023; Yan et al., 2025). In the WNT/Ca^2+^ pathway, WNT signaling induces Ca^2+^ influx, activating protein kinase C (PKC), CaMKII, and NFAT. Increased WNT5A signaling enhances NFAT activation in glioblastomas, thereby favoring immunosuppression, limiting anti-tumor immune responses, and promoting the survival of glioma stem-like cells (Lee Y. et al., 2016).

#### 2.1.2 Mutations in WNT signaling pathway components and their consequences

The oncogenic hijacking of WNT signaling is predominantly driven by mutations, with different tumors harboring different mutational landscapes. APC is a master regulator of β-catenin. Loss-of-function mutations, mostly truncating mutations, disrupt their ability to scaffold the destruction complex, allowing for β-catenin stabilization and unchecked transcriptional activation (Yan et al., 2025; Song et al., 2024). Interestingly, APC is often only partially inactivated in tumors; extreme β-catenin accumulation is avoided because it can paradoxically trigger cellular senescence. Activating β-catenin gene (*CTNNB1*) mutations primarily affect exon 3 and further impact serine/threonine residues important for β-catenin degradation. In contrast to *APC* mutations, these abnormalities are more frequently observed in liver, ovarian, and endometrial tumors, where they promote lineage-specific oncogenesis. β-catenin-mutant tumors often exhibit immune-cold phenotypes, prompting inquiries about possible obstacles to immune checkpoint blockade therapy (Yan et al., 2025; Song et al., 2024).

AXIN1 and AXIN2 are scaffolding proteins of the destruction complex responsible for efficient β-catenin degradation. Individual inactivating events are rarer than *APC* mutations but also lead to a loss of β-catenin degradation. *AXIN2* loss is particularly prominent in gastric and pancreatic cancers, where it cooperates with oncogenic *KRAS* signaling to increase WNT output (Groenewald et al., 2023; Yan et al., 2025). The established paradigm of WNT signaling as an individual tumorigenic actor is shifting. New data suggest that active WNT interacts dynamically with opposing factors, such as Hippo, Notch, and TGF-β, as determinants of cancer heterogeneity and resistance to therapy (Pelullo et al., 2019). Importantly, pharmacological inhibition of WNT signaling is still in its infancy, given the fundamental physiological roles of these pathways. However, emerging strategies, including tankyrase inhibitors, PORCN inhibitors, and antibody-based methods, have shown potential for selectively regulating its activity in a cancer-dependent way (Yadav et al., 2023; Fujita and Demizu, 2024; Zhang et al., 2025; Huang et al., 2009).

### 2.2 MiRNAs as the regulators of the WNT signaling pathway in cancer

Non-coding regulatory RNA molecules, or miRNAs, are roughly 21–23 nucleotides long, and numerous studies have extensively reviewed their biogenesis pathway (Shang et al., 2023). All organisms contain miRNAs, from the plant to the animal kingdom, which can modify a significant portion of their transcriptome (Shang et al., 2023; Zhao et al., 2018). Thousands of genes involved in many biological processes, such as cellular differentiation and development, can be controlled by miRNAs. Dysregulation of miRNAs is associated with various diseases, including cancer, autoimmune disorders, and developmental abnormalities, as a single miRNA can regulate multiple gene targets (Singh and Storey, 2021). The field of miRNA research has expanded rapidly, and miRNAs are recognized as key modulators of gene expression. Most miRNAs in the genome are located in areas distant from known genes, indicating that they belong to an independent transcription unit with their promoters. However, a small percentage of miRNAs originate from the intronic regions of pre-mRNAs transcribed from protein-coding genomic sequences, indicating their dependence on mRNA splicing mechanisms and the promoter of the corresponding gene (Shang et al., 2023). Additionally, the genome contains clusters of several miRNA genes, which suggests that they are transcriptionally multi-cistronic primary transcripts (Shang et al., 2023).

While WNT signaling tightly regulates miR production, dysregulation of miR causes constitutively active WNT activity in cancer (Peng et al., 2017; Song et al., 2015). Anton et al. identified 38 potential miRNAs after screening 470 miRNAs in a cell-based test using human HEK293 cells to determine those that regulate the activity of the WNT pathway (Anton et al., 2011). By targeting WNT ligands and receptors, as well as β-catenin-interacting complexes, transcription factors, and various components of the WNT signaling pathway, miRs can activate or repress the WNT pathway at multiple levels. WNT activation, on the other hand, raises miR expression by binding β-catenin to TCF/LEF, which subsequently binds to promoter sites to trigger transcription (Peng et al., 2017; Song et al., 2015). Additionally, several miRs and WNT signaling components have mutual feedback loops. In breast cancer cells, the WNT pathway and miR-218 form a positive feedback loop that promotes osteoblast development and the aberrant expression of osteoblastic genes (Hassan et al., 2012). During osteogenesis, miR-218 targets three WNT signaling inhibitors (sFRP2, DKK2, and sclerostin) to activate the WNT pathway. Active WNT signaling also induces the expression of miR-218, establishing a positive feedback loop (Hassan et al., 2012). In CRC stem cells, SNAIL activates the expression of miR-146a in a way dependent on β-catenin. MiR-146a then targets Numb to stabilize β-catenin (Hwang et al., 2014). A feedback loop guides symmetric cell division by activating the WNT pathway. WNT-dependent transcription triggers the production of miR-372 and miR-373, which subsequently target WNT-targeting inhibitors, such as DKK1, thereby activating the WNT/β-catenin signaling pathway (Zhou et al., 2012). It has also been noted that the miR and WNT pathways are mutually inhibited. The WNT pathway, which negatively controls miR-122 expression, is inhibited by miR-122 in glioma cells (Faramin Lashkarian et al., 2023). The WNT pathway and miR-101 develop a reciprocal inhibitory interaction in CRC. β-catenin nuclear accumulation is significantly hampered by overexpression of miR-101, whereas the WNT pathway activity suppresses miR-101 production (Strillacci et al., 2013). Furthermore, a negative feedback loop between the WNT signaling pathway and miR was discovered. MiR-483-3p targets β-catenin, which causes miR-483-3p to be expressed. In healthy cells, they create a negative feedback loop, but if β-catenin has an activating mutation, this loop is rendered inactive (Veronese et al., 2011). In conclusion, oncogenesis is driven by the reciprocal causation of WNT activation and miR-mediated gene regulation.

Having explored the mechanistic links between WNT signaling and miRNAs, we now transition into the therapeutic potential of flavonoids in modulating this axis. The following section outlines how flavonoids can suppress oncogenic miRNAs, upregulate tumor-suppressor miRNAs, and ultimately inhibit the WNT pathway, thereby providing a multifaceted strategy to restrict tumor progression.

## 3 Flavonoids as modulators of WNT signaling/miRNA complexes

**FIGURE 1:**
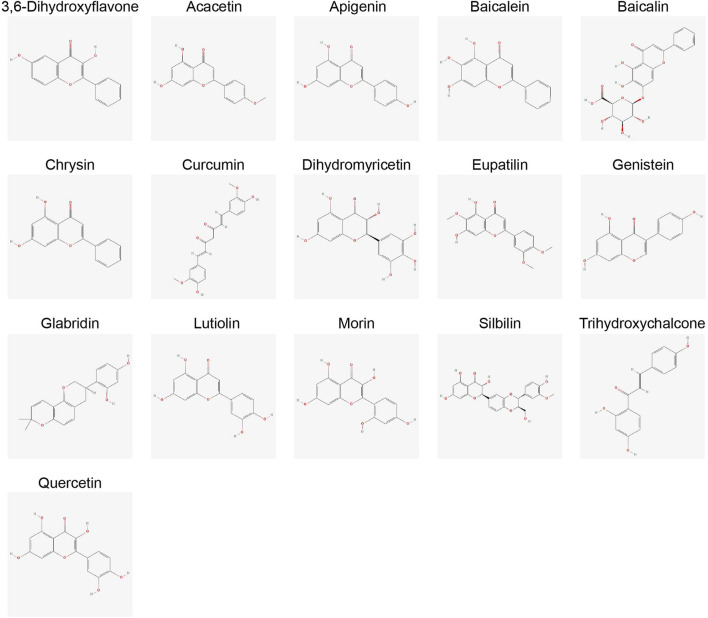
Chemical structures of key flavonoids discussed in this review. The chemical structures were obtained from the PubChem database.

**FIGURE 2:**
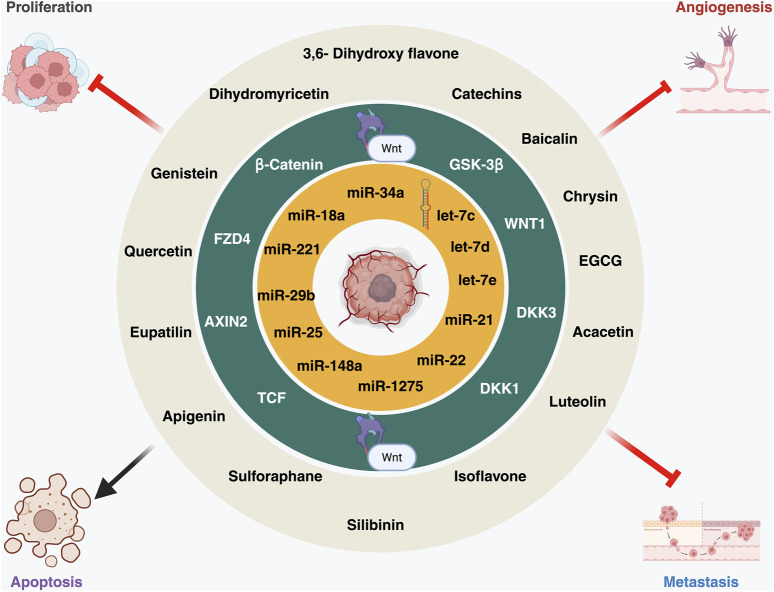
A schematic representation of this triad: flavonoids (outer circle) modulating key WNT pathway components (middle circle) and microRNAs (miRNAs) (inner circle) involved in proliferation, angiogenesis, metastasis, and apoptosis. The image was created with BioRender.com.

Building upon our understanding of miRNA-mediated WNT regulation, this section examines how flavonoids strategically influence both components, reinforcing their combined therapeutic promise. Flavonoids are polyphenolic secondary metabolites found in fruits, vegetables, and plant-based diets, known for their anti-inflammatory, antioxidant, and cancer-fighting properties (Figure 1) (Pyo et al., 2024). To exert their anticancer effects, these compounds inhibit angiogenesis, induce apoptosis, and regulate miRNAs in cancer. According to epidemiological research, a diet high in flavonoids reduces the incidence of cancers such as breast, colon, and prostate cancer by influencing important cell signaling pathways like MAPK, PI3K/AKT, and WNT/β-catenin (Figure 2) (Filippi et al., 2025; Pandey et al., 2023). This section examines the suppression of tumor progression by flavonoids through modulation of WNT/β-catenin signaling and miRNA regulation. Moreover, miRNAs play a crucial role in tumor growth, metastasis, and the development of drug resistance. By downregulating oncogenic miRNAs (oncomiRs) and increasing tumor-suppressor miRNAs, flavonoids function as dual modulators of miRNAs, re-establishing cellular equilibrium.

### 3.1 Flavonoids modulating oncogenic miRNAs

OncomiRs control genes involved in cell proliferation, metastasis, resistance to apoptosis, and chemoresistance, which are crucial for cancer progression (Pandima Devi et al., 2017; Varghese et al., 2020). These miRNAs promote carcinogenesis by targeting tumor suppressor genes or enhancing oncogenic pathways (Table 1) (Bansode et al., 2016). One of the well-known oncomiRs is miR-21, which is frequently overexpressed in various cancers, including those of the brain, breast, liver, lung, and gastrointestinal tract (Helen et al., 2024). By targeting *PTEN* and *PDCD4*, miR-21 promotes uncontrolled cell proliferation and resistance to apoptosis, contributing to cancer progression (Levenson, 2022; Izzo et al., 2020).

**TABLE 1:** A brief list of Flavonoids Targeting microRNA (miRNA) and WNT Signaling Pathways in Cancer Cell Lines.

SerialNo.	Flavonoid	Affected miRNA	Mechanism	Cancer cell lines	References
1	3,6-Dihydroxyflavone	miR-21 ↓ miR-34a ↑	Reduces DNMT1 expression Inhibits PI3K/Akt/mTOR pathway	Breast cancer (MCF-7, MDA MB-453)	Peng et al. (2015)
2	Anthocyanin	miR-34a ↑	Delphinidin increases miR-34a levels, decreases β-catenin & GSK-3β expression	Breast cancer (MCF-7, MDA MB-231, MDA MB-453)	Han et al. (2019)
		miR-483-3p ↓	Black raspberry anthocyanin inhibits proliferation and migration of cells by targeting DKK3 (WNT signaling); Tumor formation in mice	Colorectal cancer (HCT116, HT29, LoVo, SW480)	(Guo et al., 2020)
3	Baicalein	miR-3127-5p ↓	lncRNA and baicalein controls the miR-3127-5p/FZD4/WNT/β-catenin axis	Cervical cancer (SiHa, HeLa)	Yu et al. (2021)
		miR-25 ↑	Baicalein increases miR-25 & GSK-3β expression, decreases β-catenin and AXIN2 expression	Osteosarcoma (Saos-2)	Örenlili Yaylagül and Ülger (2020)
		Multiple miRNAs	Reduces cell proliferation and induces S and G2/M phase arrest; Targets WNT signaling	Hepatocellular carcinoma (Bel-7402, Hep3B)	(Bie et al., 2017)
		miR-217 ↑	Inhibits proliferation, induces apoptosis, enhances expression of DKK1, reduces expression of β-catenin, and c-MYC	Colorectal cancer (HCT116, DLD1)	(Jia et al., 2019)
4	Chrysin	miR-21 ↓ miR-18a ↓ miR-221 ↓	PLGA-PEG nanoparticle-encapsulated chrysin inhibits cell growth	Gastric cancer (AGS)	Mohammadi et al. (2016)
5	Curcumin	miR-21 ↓	Inhibits proliferation, enhances apoptosis, and restores PDCD4 expression	Gastric cancer (MGC-803)	Qiang et al. (2019)
6	Dihydromyri-cetin	miR-21 ↓	Suppresses the PI3K/Akt pathway, upregulates PTEN	Cholangio carcinoma (HCCC9810, TFK-1)	Chen et al. (2020)
7	Eupatilin	miR-21 ↓	Induces apoptosis; inhibits migration and tumor formation in mice by targeting YAP1	Renal cancer (786-O, Caki-2)	Zhong et al. (2019)
8	Genistein	miR-1260b ↓	Genistein induces apoptosis; inhibits invasion, proliferation, and TCF reporter activity	Prostate cancer (PC-3, DU-145)	Chiyomaru et al. (2013a)
				Renal cancer (A-498, 786-O, Caki-2)	Hirata et al. (2013)
		miR-574-3p ↑	Reduces cell proliferation, migration and invasion; induces apoptosis; Targets WNT signaling molecules such as WNT5A and RAC1	Prostate cancer (PC3, DU145)	(Chiyomaru et al., 2013b)
9	Glabridin	miR-148a ↑	Inhibits angiogenesis by reducing WNT1, β-catenin, and LEF/TCF4 activation	Breast cancer (MDA MB-231, Hs-578T)	(Mu et al., 2017)
10	Isoliquiritige-nin	miR-32 ↓	Induces apoptosis; inhibits proliferation, migration, & invasion; inhibits tumor formation in mice; decreases MMP2 & MMP9 expression; Inhibits WNT signaling pathway	Nasopharyngeal carcinoma (C666-1, CNE2)	Wang et al. (2019a)
11	Morin	miR-216a ↑	Reduces cell proliferation & stemness related subpopulation; reduces tumor formation in mice; decreases CD20, CD44 and CD133 expression; Inhibits WNT3A expression	Melanoma (MV3, M14)	(Hu et al., 2018)
12	Quercetin	miR-22 ↑	Induces apoptosis; inhibits cell proliferation and tumor formation in mice; decreases β-catenin and WNT1 expression; Inhibits WNT signalling pathway	Oral squamous cell carcinoma (Tca8113, SAS)	(Zhang et al., 2019a)

Besides miR-21, several other oncogenic miRNAs have been linked to cancer, particularly those associated with the WNT/β-catenin signaling pathway, a key promoter of cancer development and metastasis (Pandey et al., 2023; Amado et al., 2011). One well-characterized oncomiR, miR-155, promotes cancer progression by downregulating tumor suppressors such as SOCS1 (Suppressor of Cytokine Signaling 1) and RAD51 (DNA repair protein). This results in enhanced cell survival, immune evasion, and genomic instability, contributing to tumor growth and progression (Zadeh et al., 2016; Adinew et al., 2021). Similarly, miR-27a has been implicated in cancer promotion by facilitating the epithelial-mesenchymal transition (EMT), a critical step in cancer metastasis (Yoshioka et al., 2022).

Flavonoids exhibit anticancer activity by modulating miRNAs, particularly the oncogenic miR-21. Overexpression of miR-21 has been reported in various cancer types, where it promotes metastasis, inhibits apoptosis, drives tumor growth, and induces drug resistance (Tuli et al., 2023; Yoshioka et al., 2022). Several others and we have critically reviewed flavonoids that exhibit anticancer effects by regulating miRNAs, highlighting their potential as signature molecules for cancer therapy (Tuli et al., 2023).

### 3.2 Flavonoids and upregulation of tumor-suppressor miRNAs

Tumor-suppressor miRNAs play a crucial role in preventing the spread of malignancies. These small, non-coding RNA molecules act upon oncogenes and modulate signaling pathways to control key cellular processes such as differentiation, proliferation, and apoptosis (Menon et al., 2022). However, their downregulation can lead to uncontrolled cell growth, resistance to apoptosis, and an increased propensity for metastasis (Gambari et al., 2016). Among the well-studied miRNAs, the let-7 family and miR-34a have been implicated as crucial modulators of carcinogenic processes (Zhang L. et al., 2019; Lee H. et al., 2016). MiR-34a targets several oncogenes, including MYC and BCL2, thereby inducing apoptosis and inhibiting cancer cell survival (Werner et al., 2017; Zhai et al., 2020). Research has shown that flavonoids, such as xanthomicrol, can upregulate miR-34a expression, thereby reducing the expression of oncogenic miRNAs, including miR-21 and miR-27 (Poormolaie et al., 2023). This leads to increased apoptosis and reduced angiogenesis, highlighting the potential of flavonoid-based cancer therapies (Helen et al., 2024; Imani et al., 2018).

K-ras and Myc are selectively targeted oncogenes often overexpressed in aggressive cancers. The let-7 family of miRNAs also exhibits tumor-suppressive function by regulating these oncogenes (Appari et al., 2014). Quercetin has been shown to significantly upregulate let-7c expression in pancreatic ductal adenocarcinoma (PDAC), leading to activation of the Notch inhibitor Numbl, suppression of Notch signaling, and a reduction in tumor growth (Nwaeburu et al., 2016). Additionally, studies have found that the administration of flavonoids, such as isoflavones and 3,3′-diindolylmethane (DIM), to gemcitabine-resistant pancreatic cancer cells reactivates the expression of let-7b, let-7c, let-7d, and let-7e. This reactivation reverses the EMT, a critical step in cancer metastasis (Li et al., 2009).

### 3.3 Combination therapies using flavonoids and targeting miRNA-WNT signaling

**FIGURE 3:**
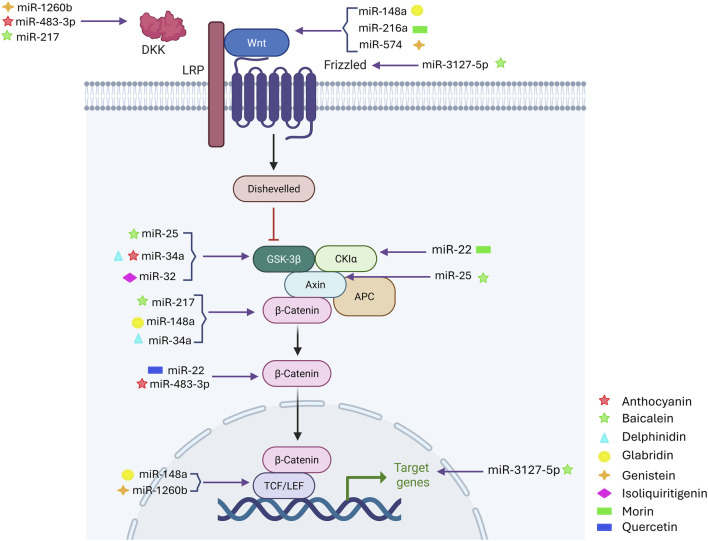
Combination Therapies Targeting the miRNA-WNT-Flavonoid Triad. Overview of combination therapeutic strategies employing flavonoids, microRNAs (miRNAs), and WNT signaling modulation. This schematic illustrates how combined approaches using specific flavonoids and miRNAs enhance therapeutic efficacy by simultaneously targeting specific components of the oncogenic WNT pathway. The image was created with BioRender.com.

Integrating flavonoid-based therapies with miRNA-WNT signaling modulation presents a promising multi-targeted strategy for treating aggressive malignancies (Figure 3) (Helen et al., 2024). The aberrant activation of the WNT/β-catenin pathway is strongly linked to tumor progression, EMT, metastasis, and therapy resistance, with dysregulated miRNAs playing a key regulatory role. Flavonoids, including apigenin, fisetin, quercetin, and luteolin, have potent anticancer effects by influencing miRNA expression and inhibiting oncogenic WNT signaling machinery (Almatroodi et al., 2024). Apigenin enhances miR-148a expression, which directly targets WNT10b. As a result, β-catenin translocation is reduced, and oncogene activation is suppressed, inhibiting tumor cell proliferation and invasion (Wang et al., 2021). Similarly, glabridin increases miR-148a expression in a dose-dependent manner by targeting the 3′ untranslated regions (UTRs), reducing WNT1 expression, and causing β-catenin accumulation at the membrane rather than in the cytoplasm and nucleus (Mu et al., 2017).

Preclinical studies suggest that quercetin inhibits PKC, an upstream modulator of WNT signaling, through miR-1275, leading to reduced cell migration and invasion (Shaalan et al., 2018). Luteolin downregulates miR-221 and miR-21, restoring tumor suppressor functions and enhancing apoptosis in cancer (Mishan et al., 2021). Additionally, curcumin-mediated upregulation of miR-27a targets SMAD2/4, a pathway that interacts with WNT signaling, resulting in a synergistic inhibition of tumor progression and metastasis (García-Hernández et al., 2024). In combination therapy, flavonoids have been explored as chemosensitizers, increasing the effectiveness of standard treatments (Liskova et al., 2021). Studies have shown that genistein, combined with gemcitabine, restores miR-200b expression, reversing EMT and inhibiting WNT-mediated resistance mechanisms in pancreatic cell lines (Li et al., 2009). Likewise, epigallocatechin gallate (EGCG), a flavonoid derived from green tea, suppresses WNT/β-catenin signaling by upregulating miR-29b, thereby reducing angiogenesis and tumor growth in preclinical models (Lyubitelev and Studitsky, 2023).

Despite the promising insights outlined in the previous sections, the WNT signaling, miRNAs, and flavonoids triad presents unique challenges, such as the mechanistic complexity of interactions, insufficient *in vivo* validation, and delivery issues. The following section addresses and critically evaluates these hurdles, outlining possible approaches to overcome the challenges.

### 3.4 Mechanistic insights: flavonoid-miRNA-WNT interactions in cancer

#### 3.4.1 Kinase interactions

Flavonoids exhibit notable interactions with kinases involved in WNT signaling in cancer, including CK1 and GSK3. Quercetin, a well-known flavonoid, binds to the ATP-binding pockets of kinases, directly inhibiting GSK3β (IC50 ∼2 µM), which stabilizes β-catenin and may promote cancer cell proliferation in specific contexts. Additionally, curcumin inhibits GSK3 with even greater potency (IC50 ∼66.3 nM), highlighting its significant therapeutic potential (Bustanji et al., 2009).

#### 3.4.2 Epigenetic modulation and miRNA expression

Flavonoids modulate cancer-associated gene expression through epigenetic mechanisms, influencing DNA methylation, histone modifications, and miRNA expression. Genistein inhibits DNA methyltransferases, thereby increasing tumor-suppressive miRNAs, such as miR-29 (Xie et al., 2014). EGCG inhibits histone deacetylases, elevating miR-15 levels, which reduces apoptosis resistance in cancer cells (Wang et al., 2023). Resveratrol enhances miR-126 expression through the SIRT1 and FOXO3 pathways, thereby impacting angiogenesis and cancer progression. In contrast, quercetin reduces the oncogenic miR-21, which in turn decreases inflammation and fibrosis associated with tumor development.

#### 3.4.3 ROS-mediated β-catenin degradation

Beyond the classical APC/Axin/CK1/GSK3β-mediated pathway, flavonoids promote β-catenin degradation through reactive oxygen species (ROS)-mediated mechanisms (Omori et al., 2011). ROS causes β-catenin degradation through a caspase-dependent mechanism that disrupts cell adhesion (Omori et al., 2011). This creates a feed-forward loop consisting of ROS, caspase activation, and β-catenin degradation that can induce cell death.

#### 3.4.4 Disruption of protein complexes in cancer

Certain flavonoids disrupt critical protein-protein interactions within WNT signaling. Genistein and baicalein inhibit β-catenin/TCF transcriptional complexes, reducing nuclear β-catenin accumulation and suppressing transcription of cancer-promoting genes (Park and Choi, 2010). Additionally, flavonoids like silibinin selectively inhibit WNT signaling in cancer cells harboring specific mutations (e.g., mutant *APC*), highlighting their potential for targeted therapeutic strategies (Kaur et al., 2010).

## 4 Clinical studies and translational implications

To effectively translate our mechanistic insights into clinical practice, this section critically reviews recent clinical trials involving flavonoid interventions targeting the miRNA-WNT axis, highlighting both achievements and persistent translational barriers.

### 4.1 Flavonoid clinical trials in cancer treatment

Recent clinical studies have demonstrated both the promise and limitations of applying flavonoid research to therapeutic applications, a topic we and others have discussed previously (Tuli et al., 2023; Liskova et al., 2021). For instance, a Phase I pilot trial examining the effects of resveratrol and grape powder on WNT pathway target gene expression in colonic mucosa and colon cancer found that grape powder containing 80g daily significantly suppressed WNT target gene expression in normal colonic mucosa but had a limited impact on cancer tissue (Nguyen et al., 2009). The study also revealed that resveratrol-containing grape powder achieved a significant inhibition of WNT signaling, with p < 0.001, indicating a potential role in colon cancer prevention rather than active treatment (Nguyen et al., 2009).

Recent clinical development analysis reveals that only 19 flavonoid-based drugs have received market approval globally, with natural flavonoids accounting for 52.6% of these approvals. Additionally, 36 flavonoid-based clinical candidates are currently in various phases of trials, although about 50% of antineoplastic and immunomodulating flavonoid candidates have been discontinued during clinical development (Xu et al., 2024).

### 4.2 Clinical challenges

#### 4.2.1 Bioavailability and pharmacokinetic limitations

The primary obstacle limiting the clinical translation of flavonoids is poor bioavailability due to low solubility, rapid metabolism, and limited absorption (Mir et al., 2024). Quercetin exhibits particularly challenging pharmacokinetics, with plasma concentrations reaching only 14 ng/mL after oral administration and undetectable levels after 5 hours (Joseph et al., 2022). However, novel formulation approaches have achieved 18.61-fold improvements in free quercetin bioavailability and 62.08-fold enhancement for total quercetin bioavailability compared to unformulated preparations (Joseph et al., 2022). Curcumin clinical trials face similar bioavailability challenges, with studies showing that the compound is metabolized within 20 min at physiological pH (Khosravi and Seifert, 2024). Clinical trials investigating curcumin in malignant diseases have revealed that studies with published results used twice the average dose (5.015g vs 2.54g) compared to unpublished studies. However, no relationship between increased dosage and improved bioavailability has been established (Khosravi and Seifert, 2024).

#### 4.2.2 Safety and toxicity concerns

EGCG clinical safety studies have identified significant hepatotoxicity risks that are both dose and route-dependent (Ramachandran et al., 2016). Repeated-dose studies demonstrated that the 14-day tolerable doses were 21.1 mg/kg for intraperitoneal administration and 67.8 mg/kg for oral administration (Ramachandran et al., 2016). Importantly, EGCG-induced hepatotoxicity was accompanied by increased serum lipid profiles, suggesting complex metabolic interactions. Flavonoids significantly inhibit the activity of the cytochrome P450 system, particularly CYP3A4, which metabolizes approximately 50% of prescribed drugs (Costa et al., 2021). This interaction increases the risk of drug toxicity, especially for medications with narrow therapeutic windows. Flavonoids also interact with ATP-binding cassette transporters, potentially enhancing both therapeutic effects and toxicity of co-administered drugs (Costa et al., 2021).

#### 4.2.3 Clinical trial design and standardization issues

A significant limitation in flavonoid clinical research is the lack of standardized methods for clinical assessment and quality measures to identify disease activity (Liskova et al., 2021). Most published curcumin studies in malignant diseases (50%) focus on treating therapy side effects rather than direct anticancer effects, with 86% investigating anti-inflammatory properties (Khosravi and Seifert, 2024). Clinical trials also often overlook inter-individual variability and genetic factors that influence flavonoid metabolism (Liskova et al., 2021). Additionally, the short duration of most clinical trials, which last only weeks to months, restricts the evaluation of long-term therapeutic benefits.

## 5 Role of nanotechnology-based formulations targeting WNT signaling in tumor suppression

Recognizing the clinical challenges of translating flavonoid-miRNA-WNT interactions into practical treatments, this section explores advanced nanotechnology-based formulations designed to overcome limitations in bioavailability and targeting. While the anticancer properties of flavonoids are well-documented, their clinical application is hindered by poor bioavailability and rapid degradation. To overcome these challenges, this section discusses the emerging role of nanotechnology-based delivery systems, which enhance the stability and targeting of flavonoid-miRNA formulations, thereby facilitating improved modulation of the WNT pathway *in vivo*.

Nanoformulations have shown enhanced therapeutic efficacy in treating various medical conditions, including cancer. Due to their restricted oral absorption, high molecular weights, and lipophilicity, flavonoids exhibit reduced bioavailability, leading to rapid elimination from the body (Gavas et al., 2021). However, nanoformulations comprising liposomes, polymeric nanoparticles, solid lipid nanoparticles, and transfersomes are well known to offer several therapeutic advantages in combination with various flavonoid constituents, thereby providing improved solubility, stability, tissue circulation, sustained delivery, and degradation resistance (Helen et al., 2024; Calin et al., 2002). Moreover, such nano vehicles are reported to bypass specific biological barriers to cancer, augment tumor penetration and passive accumulation (due to enhanced stability), and enhance overall anticancer efficacy (Grapa et al., 2019). Clinical studies have advanced flavonoid-miRNA therapeutics through the use of nanoformulations, thereby improving bioavailability and targeted delivery. Fisetin and luteolin-loaded nanoparticles enhance miRNA-mediated WNT inhibition while mitigating drug resistance in pancreatic and CRC models (Basu et al., 2023). Developing nanocarrier-based delivery systems for flavonoid-miRNA therapeutics holds significant promise for clinical applications, providing a precision medicine approach to overcoming therapy resistance. Another flavonoid, quercetin, has been shown to prevent tumor proliferation and invasion via the miR-146a/b pathway, upregulate tumor-suppressor miRNAs, including the Let-7 family, and initiate apoptosis (Wang D. et al., 2019). This flavonoid is also known to increase the expression of tumor suppressor miRNAs, predominantly miRNA-34a (Lou et al., 2015). By targeting multiple oncogenic pathways, particularly WNT/β-catenin and EMT-related signaling cascades, flavonoid-based combination therapies offer a promising avenue for future cancer treatment strategies. However, further translational research is necessary to optimize therapeutic efficacy in highly resistant tumors such as PDAC.

## 6 Challenges and future directions

Having explored detailed molecular interactions and clinical translation pathways, this final section discusses the remaining challenges and future strategies needed to realize the full potential of the miRNA-WNT-flavonoid therapeutic triad. This review integrates insights from WNT signaling, miRNA regulation, and flavonoid modulation, highlighting the synergistic potential of this triad in combating tumorigenesis. By bridging mechanistic understanding with therapeutic innovation, we aim to inspire further research into leveraging these interconnected pathways for more effective and targeted cancer therapies.

**FIGURE 4:**
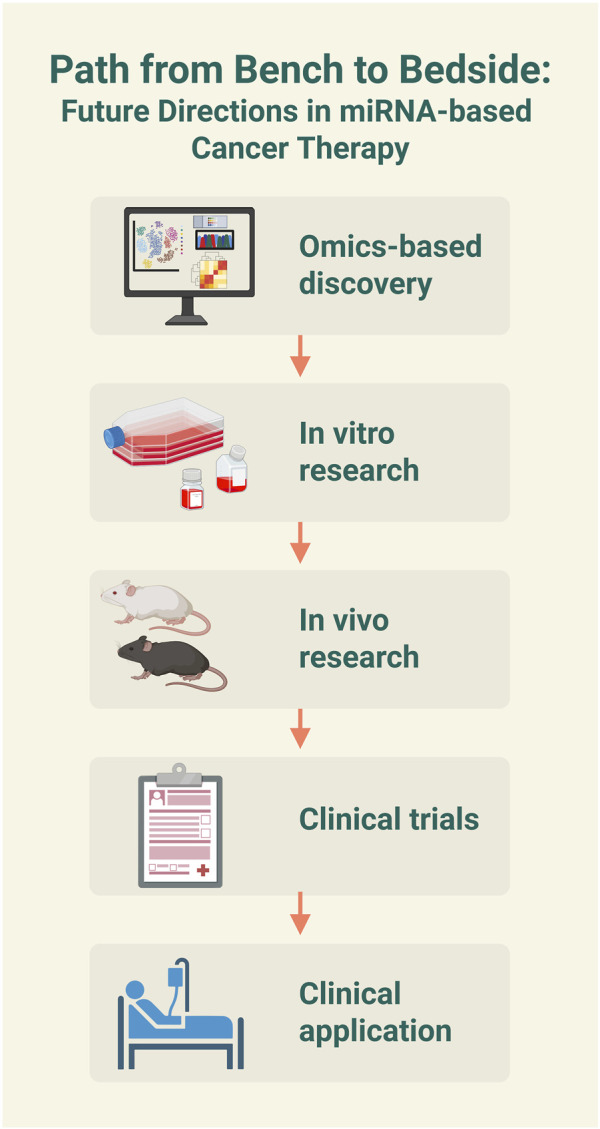
Future directions in microRNA (miRNA)-based cancer therapy. The schematic illustrates a multi-step process for developing miRNA-based treatment from the bench to the bedside. The image was created with BioRender.com.

The current research on flavonoid-mediated regulation of the WNT-miRNA axis in cancer has several limitations. The primary issue is the complex mechanism of this triad, as many unknowns remain about how flavonoids, WNT signaling, and miRNAs interact, which makes it challenging to pinpoint clear regulatory pathways. It is also possible that other biomolecules, beyond WNT and miRNA, are involved through yet-to-be-identified pathways, which complicates therapeutic applications. Furthermore, flavonoid bioavailability is often reduced due to rapid metabolic degradation and poor solubility. Therefore, advanced nanoformulation-based delivery systems that incorporate flavonoids are considered a promising approach to overcoming these challenges (Yuan et al., 2024). Another limitation is the lack of sufficient *in vivo* validation since most studies are limited to *in vitro* models, which slows down clinical testing (Figure 4). Generally, miRNA-based therapies face challenges from off-target effects, which can potentially cause unintended gene silencing and cytotoxicity. Using precise delivery systems, such as ligand-functionalized nanoparticles, is essential to improve therapeutic specificity and efficacy. To move forward, flavonoid-miRNA-WNT-targeted therapies can address these issues by employing specific miRNA modifications, advanced nanocarriers, and better *in vivo* models.

Although significant advances have been made in pharmacologic research, AI-based predictive models are expected to facilitate the discovery of flavonoid-miRNA-WNT interactions more precisely, enabling rational drug design (Luo et al., 2022). A thorough understanding of the molecular interactions of flavonoid-miRNA-WNT can also be obtained by integrating several levels of regulation of cellular processes through the use of multi-omics (Milenkovic and Ruskovska, 2023). The application of spatial transcriptomics is expected to further enhance understanding by mapping miRNA-flavonoid interactions within the tumor microenvironment, thereby uncovering spatially distinct regulatory networks (Li X. et al., 2024). Using these technologies, precision oncology can maximize flavonoid-derived miRNA therapies, stratify patients based on molecular signatures, and concurrently design customized treatment protocols, thereby enhancing clinical outcomes for therapy-resistant cancers.

## 7 Conclusion

MiRNAs represent a powerful class of gene expression regulators with profound implications in cancer pathogenesis and therapy. Their intricate interplay with WNT signaling pathways and responsiveness to natural compounds, such as flavonoids, create a versatile axis for therapeutic intervention. Flavonoid-mediated modulation of miRNA presents a novel approach to reprogram oncogenic pathways, suppress tumor progression, and overcome drug resistance. Despite significant preclinical promise, challenges such as delivery efficiency, specificity, and *in vivo* validation remain. Future integration of advanced delivery systems, spatial transcriptomics, and AI-guided modeling is critical for realizing miRNA-centered, personalized oncology. Positioning miRNAs at the heart of therapeutic strategies could redefine cancer treatment paradigms.

## References

[B1] Adinew G. M. Taka E. Mendonca P. Messeha S. S. Soliman K. F. A. (2021). The anticancer effects of flavonoids through miRNAs modulations in triple-negative breast cancer. Nutrients 13 (4), 1212. 10.3390/nu13041212 33916931 PMC8067583

[B2] Almatroodi S. A. Almatroudi A. Alharbi H. O. A. Khan A. A. Rahmani A. H. (2024). Effects and mechanisms of luteolin, a plant-based flavonoid, in the prevention of cancers via modulation of inflammation and cell signaling molecules. Molecules 29 (5), 1093. 10.3390/molecules29051093 38474604 PMC10934766

[B3] Amado N. G. Fonseca B. F. Cerqueira D. M. Neto V. M. Abreu J. G. (2011). Flavonoids: potential Wnt/beta-catenin signaling modulators in cancer. Life Sci. 89 (15-16), 545–554. 10.1016/j.lfs.2011.05.003 21635906

[B4] Anton R. Chatterjee S. S. Simundza J. Cowin P. Dasgupta R. (2011). A systematic screen for micro-RNAs regulating the canonical Wnt pathway. PLoS One 6 (10), e26257. 10.1371/journal.pone.0026257 22043311 PMC3197157

[B5] Appari M. Babu K. R. Kaczorowski A. Gross W. Herr I. (2014). Sulforaphane, quercetin and catechins complement each other in elimination of advanced pancreatic cancer by miR-let-7 induction and K-ras inhibition. Int. J. Oncol. 45 (4), 1391–1400. 10.3892/ijo.2014.2539 25017900 PMC4151818

[B6] Bansode R. R. Khatiwada J. R. Losso J. N. Williams L. L. (2016). Targeting MicroRNA in cancer using plant-based proanthocyanidins. Diseases 4 (2), 21. 10.3390/diseases4020021 28933401 PMC5456277

[B7] Basu A. Namporn T. Ruenraroengsak P. (2023). Critical review in designing plant-based anticancer nanoparticles against hepatocellular carcinoma. Pharmaceutics 15 (6), 1611. 10.3390/pharmaceutics15061611 37376061 PMC10302838

[B8] Bie B. Sun J. Li J. Guo Y. Jiang W. Huang C. (2017). Baicalein, a natural anti-cancer compound, alters MicroRNA expression profiles in bel-7402 human hepatocellular carcinoma cells. Cell Physiol. Biochem. 41 (4), 1519–1531. 10.1159/000470815 28351032

[B9] Bray F. Laversanne M. Sung H. Ferlay J. Siegel R. L. Soerjomataram I. (2024). Global cancer statistics 2022: GLOBOCAN estimates of incidence and mortality worldwide for 36 cancers in 185 countries. CA Cancer J. Clin. 74 (3), 229–263. 10.3322/caac.21834 38572751

[B10] Bustanji Y. Taha M. O. Almasri I. M. Al-Ghussein M. A. Mohammad M. K. Alkhatib H. S. (2009). Inhibition of glycogen synthase kinase by curcumin: investigation by simulated molecular docking and subsequent *in vitro*/*in vivo* evaluation. J. Enzyme Inhib. Med. Chem. 24 (3), 771–778. 10.1080/14756360802364377 18720192

[B11] Calin G. A. Dumitru C. D. Shimizu M. Bichi R. Zupo S. Noch E. (2002). Frequent deletions and down-regulation of micro- RNA genes miR15 and miR16 at 13q14 in chronic lymphocytic leukemia. Proc. Natl. Acad. Sci. U. S. A. 99 (24), 15524–15529. 10.1073/pnas.242606799 12434020 PMC137750

[B12] Chen L. Yang Z. S. Zhou Y. Z. Deng Y. Jiang P. Tan S. L. (2020). Dihydromyricetin inhibits cell proliferation, migration, invasion and promotes apoptosis via regulating miR-21 in Human Cholangiocarcinoma Cells. J. Cancer 11 (19), 5689–5699. 10.7150/jca.45970 32913463 PMC7477438

[B13] Chiyomaru T. Yamamura S. Fukuhara S. Yoshino H. Kinoshita T. Majid S. (2013a). Genistein inhibits prostate cancer cell growth by targeting miR-34a and oncogenic HOTAIR. PLoS One 8 (8), e70372. 10.1371/journal.pone.0070372 23936419 PMC3731248

[B14] Chiyomaru T. Yamamura S. Fukuhara S. Hidaka H. Majid S. Saini S. (2013b). Genistein up-regulates tumor suppressor microRNA-574-3p in prostate cancer. PLoS One 8 (3), e58929. 10.1371/journal.pone.0058929 23554959 PMC3595226

[B15] Clevers H. Nusse R. (2012). Wnt/β-catenin signaling and disease. Cell 149 (6), 1192–1205. 10.1016/j.cell.2012.05.012 22682243

[B16] Costa R. Costa Lima S. A. Gameiro P. Reis S. (2021). On the development of a cutaneous flavonoid delivery system: advances and limitations. Antioxidants (Basel) 10 (9), 1376. 10.3390/antiox10091376 34573007 PMC8472229

[B17] Emran T. B. Shahriar A. Mahmud A. R. Rahman T. Abir M. H. Siddiquee M. F. (2022). Multidrug resistance in cancer: understanding molecular mechanisms, immunoprevention and therapeutic approaches. Front. Oncol. 12, 891652. 10.3389/fonc.2022.891652 35814435 PMC9262248

[B18] Faramin Lashkarian M. Hashemipour N. Niaraki N. Soghala S. Moradi A. Sarhangi S. (2023). MicroRNA-122 in human cancers: from mechanistic to clinical perspectives. Cancer Cell Int. 23 (1), 29. 10.1186/s12935-023-02868-z 36803831 PMC9940444

[B19] Filippi A. Deculescu-Ioniță T. Hudiță A. Baldasici O. Gălățeanu B. Mocanu M. M. (2025). Molecular mechanisms of dietary compounds in cancer stem cells from solid tumors: insights into colorectal, breast, and prostate cancer. Int. J. Mol. Sci. 26 (2), 631. 10.3390/ijms26020631 39859345 PMC11766403

[B20] Fujita M. Demizu Y. (2024). Advances in the development of Wnt/β-catenin signaling inhibitors. RSC Med. Chem. 16, 984–999. 10.1039/d4md00749b PMC11647577 39691403

[B21] Gambari R. Brognara E. Spandidos D. A. Fabbri E. (2016). Targeting oncomiRNAs and mimicking tumor suppressor miRNAs: Νew trends in the development of miRNA therapeutic strategies in oncology (Review). Int. J. Oncol. 49 (1), 5–32. 10.3892/ijo.2016.3503 27175518 PMC4902075

[B22] García-Hernández A. P. Sánchez-Sánchez G. Carlos-Reyes A. López-Camarillo C. (2024). Functional roles of microRNAs in vasculogenic mimicry and resistance to therapy in human cancers: an update. Expert Rev. Clin. Immunol. 20 (8), 913–926. 10.1080/1744666x.2024.2352484 38712535

[B23] Gavas S. Quazi S. Karpiński T. M. (2021). Nanoparticles for cancer therapy: current progress and challenges. Nanoscale Res. Lett. 16 (1), 173. 10.1186/s11671-021-03628-6 34866166 PMC8645667

[B24] Grapa C. M. Mocan T. Gonciar D. Zdrehus C. Mosteanu O. Pop T. (2019). Epidermal growth factor receptor and its role in pancreatic cancer treatment mediated by nanoparticles. Int. J. Nanomedicine 14, 9693–9706. 10.2147/ijn.S226628 31849462 PMC6910098

[B25] Groenewald W. Lund A. H. Gay D. M. (2023). The role of WNT pathway mutations in cancer development and an overview of therapeutic options. Cells 12 (7), 990. 10.3390/cells12070990 37048063 PMC10093220

[B26] Guo J. Yang Z. Zhou H. Yue J. Mu T. Zhang Q. (2020). Upregulation of DKK3 by miR-483-3p plays an important role in the chemoprevention of colorectal cancer mediated by black raspberry anthocyanins. Mol. Carcinog. 59 (2), 168–178. 10.1002/mc.23138 31763724

[B27] Han B. Peng X. Cheng D. Zhu Y. Du J. Li J. (2019). Delphinidin suppresses breast carcinogenesis through the HOTAIR/microRNA-34a axis. Cancer Sci. 110 (10), 3089–3097. 10.1111/cas.14133 31325197 PMC6778627

[B28] Hassan M. Q. Maeda Y. Taipaleenmaki H. Zhang W. Jafferji M. Gordon J. A. (2012). miR-218 directs a Wnt signaling circuit to promote differentiation of osteoblasts and osteomimicry of metastatic cancer cells. J. Biol. Chem. 287 (50), 42084–42092. 10.1074/jbc.M112.377515 23060446 PMC3516754

[B29] Helen H. Gunawan M. C. Halim P. Dinata M. R. Ahmed A. Dalimunthe A. (2024). Flavonoids as modulators of miRNA expression in pancreatic cancer: pathways, mechanisms, and therapeutic potential. Biomed. Pharmacother. 179, 117347. 10.1016/j.biopha.2024.117347 39241569

[B30] Hirata H. Ueno K. Nakajima K. Tabatabai Z. L. Hinoda Y. Ishii N. (2013). Genistein downregulates onco-miR-1260b and inhibits Wnt-signalling in renal cancer cells. Br. J. Cancer 108 (10), 2070–2078. 10.1038/bjc.2013.173 23591200 PMC3670482

[B31] Hu J. Guo X. Yang L. (2018). Morin inhibits proliferation and self-renewal of CD133(+) melanoma cells by upregulating miR-216a. J. Pharmacol. Sci. 136 (3), 114–120. 10.1016/j.jphs.2018.02.003 29496393

[B32] Huang S.-M. A. Mishina Y. M. Liu S. Cheung A. Stegmeier F. Michaud G. A. (2009). Tankyrase inhibition stabilizes axin and antagonizes Wnt signalling. Nature 461 (7264), 614–620. 10.1038/nature08356 19759537

[B33] Hushmandi K. Alimohammadi M. Heiat M. Hashemi M. Nabavi N. Tabari T. (2025). Targeting Wnt signaling in cancer drug resistance: insights from pre-clinical and clinical research. Pathol. Res. Pract. 267, 155837. 10.1016/j.prp.2025.155837 39954370

[B34] Hwang W. L. Jiang J. K. Yang S. H. Huang T. S. Lan H. Y. Teng H. W. (2014). MicroRNA-146a directs the symmetric division of Snail-dominant colorectal cancer stem cells. Nat. Cell Biol. 16 (3), 268–280. 10.1038/ncb2910 24561623

[B35] Imani S. Wu R. C. Fu J. (2018). MicroRNA-34 family in breast cancer: from research to therapeutic potential. J. Cancer 9 (20), 3765–3775. 10.7150/jca.25576 30405848 PMC6216011

[B36] Izzo S. Naponelli V. Bettuzzi S. (2020). Flavonoids as epigenetic modulators for prostate cancer prevention. Nutrients 12 (4), 1010. 10.3390/nu12041010 32268584 PMC7231128

[B37] Jia Y. Chen L. Guo S. Li Y. (2019). Baicalin induced colon cancer cells apoptosis through miR-217/DKK1-mediated inhibition of Wnt signaling pathway. Mol. Biol. Rep. 46 (2), 1693–1700. 10.1007/s11033-019-04618-9 30737617

[B38] Joseph A. Shanmughan P. Balakrishnan A. Maliakel B. M K. I. (2022). Enhanced bioavailability and pharmacokinetics of a natural self-emulsifying reversible hybrid-hydrogel system of quercetin: a randomized double-blinded comparative crossover study. ACS Omega 7 (50), 46825–46832. 10.1021/acsomega.2c05929 36570285 PMC9774360

[B39] Kaur M. Velmurugan B. Tyagi A. Agarwal C. Singh R. P. Agarwal R. (2010). Silibinin suppresses growth of human colorectal carcinoma SW480 cells in culture and xenograft through down-regulation of beta-catenin-dependent signaling. Neoplasia 12 (5), 415–424. 10.1593/neo.10188 20454513 PMC2864479

[B40] Khosravi M. A. Seifert R. (2024). Clinical trials on curcumin in relation to its bioavailability and effect on malignant diseases: critical analysis. Naunyn Schmiedeb. Arch. Pharmacol. 397 (5), 3477–3491. 10.1007/s00210-023-02825-7 PMC11074217 37966571

[B41] Kilmister E. J. Koh S. P. Weth F. R. Gray C. Tan S. T. (2022). Cancer metastasis and treatment resistance: mechanistic insights and therapeutic targeting of cancer stem cells and the tumor microenvironment. Biomedicines 10 (11), 2988. 10.3390/biomedicines10112988 36428556 PMC9687343

[B42] Lee Y. Lee J.-K. Ahn S. H. Lee J. Nam D.-H. (2016a). WNT signaling in glioblastoma and therapeutic opportunities. Lab. Investig. 96 (2), 137–150. 10.1038/labinvest.2015.140 26641068

[B43] Lee H. Han S. Kwon C. S. Lee D. (2016b). Biogenesis and regulation of the let-7 miRNAs and their functional implications. Protein Cell 7 (2), 100–113. 10.1007/s13238-015-0212-y 26399619 PMC4742387

[B44] Lei Y. Chen L. Zhang G. Shan A. Ye C. Liang B. (2020). MicroRNAs target the Wnt/β‑catenin signaling pathway to regulate epithelial‑mesenchymal transition in cancer (Review). Oncol. Rep. 44 (4), 1299–1313. 10.3892/or.2020.7703 32700744 PMC7448411

[B45] Levenson A. S. (2022). Dietary stilbenes as modulators of specific miRNAs in prostate cancer. Front. Pharmacol. 13, 970280. 10.3389/fphar.2022.970280 36091792 PMC9449421

[B46] Li Y. VandenBoom T. G. 2nd Kong D. Wang Z. Ali S. Philip P. A. (2009). Up-regulation of miR-200 and let-7 by natural agents leads to the reversal of epithelial-to-mesenchymal transition in gemcitabine-resistant pancreatic cancer cells. Cancer Res. 69 (16), 6704–6712. 10.1158/0008-5472.Can-09-1298 19654291 PMC2727571

[B47] Li H. Li X. Du W. (2024a). Interplay between Wnt signaling molecules and exosomal miRNAs in breast cancer (Review). Oncol. Rep. 52 (2), 107. 10.3892/or.2024.8766 38940326 PMC11234250

[B48] Li X. Li B. Gu S. Pang X. Mason P. Yuan J. (2024b). Single-cell and spatial RNA sequencing reveal the spatiotemporal trajectories of fruit senescence. Nat. Commun. 15 (1), 3108. 10.1038/s41467-024-47329-x 38600080 PMC11006883

[B49] Liskova A. Samec M. Koklesova L. Brockmueller A. Zhai K. Abdellatif B. (2021). Flavonoids as an effective sensitizer for anti-cancer therapy: insights into multi-faceted mechanisms and applicability towards individualized patient profiles. Epma J. 12 (2), 155–176. 10.1007/s13167-021-00242-5 34025826 PMC8126506

[B50] Loeb L. A. Harris C. C. (2008). Advances in chemical carcinogenesis: a historical review and prospective. Cancer Res. 68 (17), 6863–6872. 10.1158/0008-5472.Can-08-2852 18757397 PMC2583449

[B51] Lou G. Liu Y. Wu S. Xue J. Yang F. Fu H. (2015). The p53/miR-34a/SIRT1 positive feedback loop in quercetin-induced apoptosis. Cell Physiol. Biochem. 35 (6), 2192–2202. 10.1159/000374024 25896587

[B52] Luo Y. Peng L. Shan W. Sun M. Luo L. Liang W. (2022). Machine learning in the development of targeting microRNAs in human disease. Front. Genet. 13, 1088189. 10.3389/fgene.2022.1088189 36685965 PMC9845262

[B53] Lyubitelev A. Studitsky V. (2023). Inhibition of cancer development by natural plant polyphenols: molecular mechanisms. Int. J. Mol. Sci. 24 (13), 10663. 10.3390/ijms241310663 37445850 PMC10341686

[B54] Menon A. Abd-Aziz N. Khalid K. Poh C. L. Naidu R. (2022). miRNA: a promising therapeutic target in cancer. Int. J. Mol. Sci. 23 (19), 11502. 10.3390/ijms231911502 36232799 PMC9569513

[B55] Milenkovic D. Ruskovska T. (2023). Mechanistic insights into dietary (poly)phenols and vascular dysfunction-related diseases using multi-omics and integrative approaches: machine learning as a next challenge in nutrition research. Mol. Asp. Med. 89, 101101. 10.1016/j.mam.2022.101101 35728999

[B56] Mir S. A. Dar A. Hamid L. Nisar N. Malik J. A. Ali T. (2024). Flavonoids as promising molecules in the cancer therapy: an insight. Curr. Res. Pharmacol. Drug Discov. 6, 100167. 10.1016/j.crphar.2023.100167 38144883 PMC10733705

[B57] Mishan M. A. Khazeei Tabari M. A. Mahrooz A. Bagheri A. (2021). Role of microRNAs in the anticancer effects of the flavonoid luteolin: a systematic review. Eur. J. Cancer Prev. 30 (5), 413–421. 10.1097/cej.0000000000000645 33720053

[B58] Mohammadian F. Pilehvar-Soltanahmadi Y. Mofarrah M. Dastani-Habashi M. Zarghami N. (2016). Down regulation of miR-18a, miR-21 and miR-221 genes in gastric cancer cell line by chrysin-loaded PLGA-PEG nanoparticles. Artif. Cells Nanomed Biotechnol. 44 (8), 1972–1978. 10.3109/21691401.2015.1129615 26772615

[B59] Mu J. Zhu D. Shen Z. Ning S. Liu Y. Chen J. (2017). The repressive effect of miR-148a on Wnt/β-catenin signaling involved in Glabridin-induced anti-angiogenesis in human breast cancer cells. BMC Cancer 17 (1), 307. 10.1186/s12885-017-3298-1 28464803 PMC5414299

[B60] Nguyen A. V. Martinez M. Stamos M. J. Moyer M. P. Planutis K. Hope C. (2009). Results of a phase I pilot clinical trial examining the effect of plant-derived resveratrol and grape powder on Wnt pathway target gene expression in colonic mucosa and colon cancer. Cancer Manag. Res. 1, 25–37. 10.2147/cmar.s4544 21188121 PMC3004662

[B61] Nie X. Liu H. Liu L. Wang Y. D. Chen W. D. (2020). Emerging roles of wnt ligands in human colorectal cancer. Front. Oncol. 10, 1341. 10.3389/fonc.2020.01341 32923386 PMC7456893

[B62] Nwaeburu C. C. Bauer N. Zhao Z. Abukiwan A. Gladkich J. Benner A. (2016). Up-regulation of microRNA let-7c by quercetin inhibits pancreatic cancer progression by activation of Numbl. Oncotarget 7 (36), 58367–58380. 10.18632/oncotarget.11122 27521217 PMC5295436

[B63] O'Brien S. Chidiac R. Angers S. (2023). Modulation of Wnt-β-catenin signaling with antibodies: therapeutic opportunities and challenges. Trends Pharmacol. Sci. 44 (6), 354–365. 10.1016/j.tips.2023.03.008 37085400

[B64] Omori E. Matsumoto K. Ninomiya-Tsuji J. (2011). Non-canonical β-catenin degradation mediates reactive oxygen species-induced epidermal cell death. Oncogene 30 (30), 3336–3344. 10.1038/onc.2011.49 21383695 PMC3131442

[B65] Onyido E. K. Sweeney E. Nateri A. S. (2016). Wnt-signalling pathways and microRNAs network in carcinogenesis: experimental and bioinformatics approaches. Mol. Cancer 15 (1), 56. 10.1186/s12943-016-0541-3 27590724 PMC5010773

[B66] Örenlili Yaylagül E. Ülger C. (2020). The effect of baicalein on Wnt/β-catenin pathway and miR-25 expression in Saos-2 osteosarcoma cell line. Turk J. Med. Sci. 50 (44), 1168–1179. 10.3906/sag-2001-161 32283909 PMC7379426

[B67] Pandey P. Khan F. Seifeldin S. A. Alshaghdali K. Siddiqui S. Abdelwadoud M. E. (2023). Targeting wnt/β-catenin pathway by flavonoids: implication for cancer therapeutics. Nutrients 15 (9), 2088. 10.3390/nu15092088 37432240 PMC10181252

[B68] Pandima Devi K. Rajavel T. Daglia M. Nabavi S. F. Bishayee A. Nabavi S. M. (2017). Targeting miRNAs by polyphenols: novel therapeutic strategy for cancer. Semin. Cancer Biol. 46, 146–157. 10.1016/j.semcancer.2017.02.001 28185862

[B69] Park S. Choi J. (2010). Inhibition of beta-catenin/Tcf signaling by flavonoids. J. Cell Biochem. 110 (6), 1376–1385. 10.1002/jcb.22654 20564233

[B70] Pelullo M. Zema S. Nardozza F. Checquolo S. Screpanti I. Bellavia D. (2019). Wnt, Notch, and TGF-β pathways impinge on hedgehog signaling complexity: an open window on cancer. Front. Genet. 10, 711. 10.3389/fgene.2019.00711 31552081 PMC6736567

[B71] Peng X. Chang H. Gu Y. Chen J. Yi L. Xie Q. (2015). 3,6-Dihydroxyflavone suppresses breast carcinogenesis by epigenetically regulating miR-34a and miR-21. Cancer Prev. Res. (Phila) 8 (6), 509–517. 10.1158/1940-6207.Capr-14-0357 25784176

[B72] Peng Y. Zhang X. Feng X. Fan X. Jin Z. (2017). The crosstalk between microRNAs and the Wnt/β-catenin signaling pathway in cancer. Oncotarget 8 (8), 14089–14106. 10.18632/oncotarget.12923 27793042 PMC5355165

[B73] Poormolaie N. Mohammadi M. Mir A. Asadi M. Kararoudi A. N. Vahedian V. (2023). Xanthomicrol: effective therapy for cancer treatment. Toxicol. Rep. 10, 436–440. 10.1016/j.toxrep.2023.02.008 37102154 PMC10123071

[B74] Pyo Y. Kwon K. H. Jung Y. J. (2024). Anticancer potential of flavonoids: their role in cancer prevention and health benefits. Foods 13 (14), 2253. 10.3390/foods13142253 39063337 PMC11276387

[B75] Qiang Z. Meng L. Yi C. Yu L. Chen W. Sha W. (2019). Curcumin regulates the miR-21/PTEN/Akt pathway and acts in synergy with PD98059 to induce apoptosis of human gastric cancer MGC-803 cells. J. Int. Med. Res. 47 (3), 1288–1297. 10.1177/0300060518822213 30727807 PMC6421392

[B76] Ramachandran B. Jayavelu S. Murhekar K. Rajkumar T. (2016). Repeated dose studies with pure Epigallocatechin-3-gallate demonstrated dose and route dependant hepatotoxicity with associated dyslipidemia. Toxicol. Rep. 3, 336–345. 10.1016/j.toxrep.2016.03.001 28959554 PMC5615837

[B77] Sak K. (2014). Cytotoxicity of dietary flavonoids on different human cancer types. Pharmacogn. Rev. 8 (16), 122–146. 10.4103/0973-7847.134247 25125885 PMC4127821

[B78] Shaalan Y. M. Handoussa H. Youness R. A. Assal R. A. El-Khatib A. H. Linscheid M. W. (2018). Destabilizing the interplay between miR-1275 and IGF2BPs by Tamarix articulata and quercetin in hepatocellular carcinoma. Nat. Prod. Res. 32 (18), 2217–2220. 10.1080/14786419.2017.1366478 28817968

[B79] Shang R. Lee S. Senavirathne G. Lai E. C. (2023). microRNAs in action: biogenesis, function and regulation. Nat. Rev. Genet. 24 (12), 816–833. 10.1038/s41576-023-00611-y 37380761 PMC11087887

[B80] Singh G. Storey K. B. (2021). MicroRNA cues from nature: a roadmap to decipher and combat challenges in human health and disease? Cells 10 (12), 3374. 10.3390/cells10123374 34943882 PMC8699674

[B81] Song J. L. Nigam P. Tektas S. S. Selva E. (2015). microRNA regulation of Wnt signaling pathways in development and disease. Cell Signal 27 (7), 1380–1391. 10.1016/j.cellsig.2015.03.018 25843779 PMC4437805

[B82] Song P. Gao Z. Bao Y. Chen L. Huang Y. Liu Y. (2024). Wnt/β-catenin signaling pathway in carcinogenesis and cancer therapy. J. Hematol. Oncol. 17 (1), 46. 10.1186/s13045-024-01563-4 38886806 PMC11184729

[B83] Strillacci A. Valerii M. C. Sansone P. Caggiano C. Sgromo A. Vittori L. (2013). Loss of miR-101 expression promotes Wnt/β-catenin signalling pathway activation and malignancy in colon cancer cells. J. Pathol. 229 (3), 379–389. 10.1002/path.4097 22930392

[B84] Talebi M. Farkhondeh T. Harifi-Mood M. S. Talebi M. Samarghandian S. (2023). Mechanistic features and therapeutic implications related to the MiRNAs and wnt signaling regulatory in breast cancer. Curr. Mol. Pharmacol. 16 (5), 530–541. 10.2174/1874467216666221017122105 36263474

[B85] Tuli H. S. Garg V. K. Bhushan S. Uttam V. Sharma U. Jain A. (2023). Natural flavonoids exhibit potent anticancer activity by targeting microRNAs in cancer: a signature step hinting towards clinical perfection. Transl. Oncol. 27, 101596. 10.1016/j.tranon.2022.101596 36473401 PMC9727168

[B86] Varghese E. Liskova A. Kubatka P. Mathews Samuel S. Büsselberg D. (2020). Anti-angiogenic effects of phytochemicals on miRNA regulating breast cancer progression. Biomolecules 10 (2), 191. 10.3390/biom10020191 32012744 PMC7072640

[B87] Veronese A. Visone R. Consiglio J. Acunzo M. Lupini L. Kim T. (2011). Mutated beta-catenin evades a microRNA-dependent regulatory loop. Proc. Natl. Acad. Sci. U. S. A. 108 (12), 4840–4845. 10.1073/pnas.1101734108 21383185 PMC3064338

[B88] Wang T. T. Chen Z. Z. Xie P. Zhang W. J. Du M. Y. Liu Y. T. (2019a). Isoliquiritigenin suppresses the proliferation and induced apoptosis via miR-32/LATS2/Wnt in nasopharyngeal carcinoma. Eur. J. Pharmacol. 856, 172352. 10.1016/j.ejphar.2019.04.033 31004603

[B89] Wang D. Sun-Waterhouse D. Li F. Xin L. Li D. (2019b). MicroRNAs as molecular targets of quercetin and its derivatives underlying their biological effects: a preclinical strategy. Crit. Rev. Food Sci. Nutr. 59 (14), 2189–2201. 10.1080/10408398.2018.1441123 29446655

[B90] Wang S. M. Yang P. W. Feng X. J. Zhu Y. W. Qiu F. J. Hu X. D. (2021). Apigenin inhibits the growth of hepatocellular carcinoma cells by affecting the expression of microRNA transcriptome. Front. Oncol. 11, 657665. 10.3389/fonc.2021.657665 33959508 PMC8095173

[B91] Wang C. Bai M. Sun Z. Yao N. Zhang A. Guo S. (2023). Epigallocatechin-3-gallate and cancer: focus on the role of microRNAs. Cancer Cell Int. 23 (1), 241. 10.1186/s12935-023-03081-8 37838685 PMC10576883

[B92] Werner T. V. Hart M. Nickels R. Kim Y. J. Menger M. D. Bohle R. M. (2017). MiR-34a-3p alters proliferation and apoptosis of meningioma cells *in vitro* and is directly targeting SMAD4, FRAT1 and BCL2. Aging (Albany NY) 9 (3), 932–954. 10.18632/aging.101201 28340489 PMC5391240

[B93] Xie Q. Bai Q. Zou L. Y. Zhang Q. Y. Zhou Y. Chang H. (2014). Genistein inhibits DNA methylation and increases expression of tumor suppressor genes in human breast cancer cells. Genes Chromosom. Cancer 53 (5), 422–431. 10.1002/gcc.22154 24532317

[B94] Xu K. Ren X. Wang J. Zhang Q. Fu X. Zhang P. C. (2024). Clinical development and informatics analysis of natural and semi-synthetic flavonoid drugs: a critical review. J. Adv. Res. 63, 269–284. 10.1016/j.jare.2023.11.007 37949300 PMC11380023

[B95] Yadav V. Jobe N. Mehdawi L. Andersson T. (2021). Targeting oncogenic WNT signalling with WNT signalling-derived peptides. Handb. Exp. Pharmacol. 269, 279–303. 10.1007/164_2021_528 34455485

[B96] Yadav V. Islam R. Tuli H. S. (2023). Patent landscape highlighting double-edged scaffold of a WNT5A-agonizing peptide, Foxy5. Pharm. Pat. Anal. 12 (2), 69–77. 10.4155/ppa-2022-0037 37078761

[B97] Yan Y. Gong Y. Liang X. Xiong Q. Lin J. Wu Y. (2025). Decoding β-catenin associated protein-protein interactions: emerging cancer therapeutic opportunities. Biochim. Biophys. Acta Rev. Cancer 1880 (1), 189232. 10.1016/j.bbcan.2024.189232 39643250

[B98] Yoshioka Y. Ohishi T. Nakamura Y. Fukutomi R. Miyoshi N. (2022). Anti-cancer effects of dietary polyphenols via ROS-mediated pathway with their modulation of MicroRNAs. Molecules 27 (12), 3816. 10.3390/molecules27123816 35744941 PMC9227902

[B99] Yu X. Xia J. Cao Y. Tang L. Tang X. Li Z. (2021). SNHG1 represses the anti-cancer roles of baicalein in cervical cancer through regulating miR-3127-5p/FZD4/Wnt/β-catenin signaling. Exp. Biol. Med. (Maywood) 246 (1), 20–30. 10.1177/1535370220955139 32883110 PMC7798002

[B100] Yuan D. Guo Y. Pu F. Yang C. Xiao X. Du H. (2024). Opportunities and challenges in enhancing the bioavailability and bioactivity of dietary flavonoids: a novel delivery system perspective. Food Chem. 430, 137115. 10.1016/j.foodchem.2023.137115 37566979

[B101] Zadeh M. M. Motamed N. Ranji N. Majidi M. Falahi F. (2016). Silibinin-induced apoptosis and downregulation of MicroRNA-21 and MicroRNA-155 in MCF-7 human breast cancer cells. J. Breast Cancer 19 (1), 45–52. 10.4048/jbc.2016.19.1.45 27066095 PMC4822106

[B102] Zhai L. Zhao Y. Liu Z. Wu J. Lin L. (2020). mRNA expression profile analysis reveals a C-MYC/miR-34a pathway involved in the apoptosis of diffuse large B-cell lymphoma cells induced by Yiqichutan treatment. Exp. Ther. Med. 20 (3), 2157–2165. 10.3892/etm.2020.8940 32765691 PMC7401774

[B103] Zhan T. Rindtorff N. Boutros M. (2017). Wnt signaling in cancer. Oncogene 36 (11), 1461–1473. 10.1038/onc.2016.304 27617575 PMC5357762

[B104] Zhang C. Hao Y. Sun Y. Liu P. (2019a). Quercetin suppresses the tumorigenesis of oral squamous cell carcinoma by regulating microRNA-22/WNT1/β-catenin axis. J. Pharmacol. Sci. 140 (2), 128–136. 10.1016/j.jphs.2019.03.005 31257059

[B105] Zhang L. Liao Y. Tang L. (2019b). MicroRNA-34 family: a potential tumor suppressor and therapeutic candidate in cancer. J. Exp. Clin. Cancer Res. 38 (1), 53. 10.1186/s13046-019-1059-5 30717802 PMC6360685

[B106] Zhang J. Guo H. Gong C. Shen J. Jiang G. Liu J. (2025). Therapeutic targets in the Wnt signaling pathway: treating cancer with specificity. Biochem. Pharmacol. 236, 116848. 10.1016/j.bcp.2025.116848 40049295

[B107] Zhao Y. Cong L. Lukiw W. J. (2018). Plant and animal microRNAs (miRNAs) and their potential for inter-kingdom communication. Cell Mol. Neurobiol. 38 (1), 133–140. 10.1007/s10571-017-0547-4 28879580 PMC11482019

[B108] Zhao H. Ming T. Tang S. Ren S. Yang H. Liu M. (2022). Wnt signaling in colorectal cancer: pathogenic role and therapeutic target. Mol. Cancer 21 (1), 144. 10.1186/s12943-022-01616-7 35836256 PMC9281132

[B109] Zhong W. Wu Z. Chen N. Zhong K. Lin Y. Jiang H. (2019). Eupatilin inhibits renal cancer growth by downregulating MicroRNA-21 through the activation of YAP1. Biomed. Res. Int. 2019, 5016483. 10.1155/2019/5016483 31179326 PMC6507163

[B110] Zhou A. D. Diao L. T. Xu H. Xiao Z. D. Li J. H. Zhou H. (2012). β-Catenin/LEF1 transactivates the microRNA-371-373 cluster that modulates the Wnt/β-catenin-signaling pathway. Oncogene 31 (24), 2968–2978. 10.1038/onc.2011.461 22020335

